# Robust Vehicle Speed Measurement Based on Feature Information Fusion for Vehicle Multi-Characteristic Detection

**DOI:** 10.3390/e23070910

**Published:** 2021-07-17

**Authors:** Lei Yang, Jianchen Luo, Xiaowei Song, Menglong Li, Pengwei Wen, Zixiang Xiong

**Affiliations:** 1 School of Electronic and Information, Zhongyuan University of Technology, Zhengzhou 450007, China; 2018006080@zut.edu.cn (J.L.); pwwen@zut.edu.cn (P.W.); 2 Dongjing Avenue Campus, Kaifeng University, Kaifeng 475004, China; 3 School of Electrical and Information Engineering, Tianjin University, Tianjin 300072, China; limenglong@tju.edu.cn; 4 Department of Electrical and Computer Engineering, Texas A&M University, College Station, TX 77843, USA; zx@ece.tamu.edu

**Keywords:** feature information fusion, cross-channel interaction, YOLOv4, ECA, vehicle speed measurement

## Abstract

A robust vehicle speed measurement system based on feature information fusion for vehicle multi-characteristic detection is proposed in this paper. A vehicle multi-characteristic dataset is constructed. With this dataset, seven CNN-based modern object detection algorithms are trained for vehicle multi-characteristic detection. The FPN-based YOLOv4 is selected as the best vehicle multi-characteristic detection algorithm, which applies feature information fusion of different scales with both rich high-level semantic information and detailed low-level location information. The YOLOv4 algorithm is improved by combing with the attention mechanism, in which the residual module in YOLOv4 is replaced by the ECA channel attention module with cross channel interaction. An improved ECA-YOLOv4 object detection algorithm based on both feature information fusion and cross channel interaction is proposed, which improves the performance of YOLOv4 for vehicle multi-characteristic detection and reduces the model parameter size and FLOPs as well. A multi-characteristic fused speed measurement system based on license plate, logo, and light is designed accordingly. The system performance is verified by experiments. The experimental results show that the speed measurement error rate of the proposed system meets the requirement of the China national standard GB/T 21555-2007 in which the speed measurement error rate should be less than 6%. The proposed system can efficiently enhance the vehicle speed measurement accuracy and effectively improve the vehicle speed measurement robustness.

## 1. Introduction

Vehicle speed measurement is one of the most important tasks of intelligent traffic monitoring system [1,2,3,4]. It helps to monitor speeding behavior and improve road safety. Common vehicle speed measurement methods can be divided into intrusive and nonintrusive. The intrusive method uses inductive loop detectors (ILDs) embedded in the road to measure the average vehicle speed, which is difficult to install and maintain due to the damage to road [5]. The nonintrusive speed measurement methods include Radar [6], Lidar [7], and video-based speed measurement systems [8]. The Radar method uses Doppler effect produced by the relative motion between vehicle and radar equipment (fixed or mobile) to measure the vehicle speed [6]. The Lidar method uses a laser to measure the distance from a fixed lidar equipment to vehicle twice within a specific time interval, and calculates the average vehicle speed [7]. Both methods transmit signals, which are easy to be detected and hard to make secret measurements. Video-based speed measurement methods can be further divided into two categories: monocular 2D video-based method and binocular 3D video-based method [9]. The monocular 2D video-based methods [8,10,11] use the perspective projection relationship of 2D imaging to estimate the distance traveled by the vehicle within a fixed frame interval, thereby calculate the vehicle speed. Due to the perspective projection relationship, they can only accurately measure the speed of vehicle in straight-line motion, but not in curved motion. The binocular 3D video-based methods [12,13,14] use the stereo imaging principle of the binocular camera, which can directly calculate the distance traveled by the vehicle within a fixed frame interval, thereby calculate the vehicle speed. However, the existing methods still have disadvantages such as low object detection efficiency and lack of intelligence, which need to be further improved.

To overcome the shortcomings of the existing methods, a binocular stereovision-based vehicle speed measurement system is proposed in [14]. The system consists of three parts: vehicle characteristic detection, vehicle tracking and stereo matching, and vehicle speed and trajectory measurement. The system uses a SSD (Single-Shot Multibox Detector) network [15] optimized for license plate detection to efficiently detect the license plate in binocular stereo video, performs fast stereo matching [16,17] in the detected left-view and right-view license plate areas, calculates the 3D coordinates of the matching point pairs, eliminates the abnormal points and selects the point closest to the license plate center as the speed measurement point of the current frame, and calculates the vehicle speed by dividing the distance between the speed measurement points in consecutive frames by the frame interval. This system is of low cost and high accuracy. It can realize nonintrusive and secret vehicle speed measurement, and can simultaneously measure the speed of multiple vehicles in different motions and on multiple lanes. The system is based on vehicle license plate detection, and the detection network is optimized for license plate detection. Moreover, the system designed is also based on the premise that the license plate characteristic of the vehicle can be accurately detected. However, vehicles license plate violation often exists, including no license plate, deliberately shielding or blurring license plate, faking license plate, etc. [18]. According to traffic statistical reports [19,20,21,22], license plate violation accounts for a relatively high proportion of traffic violation, which poses a challenge to the binocular stereovision-based vehicle speed measurement system with license plate in [14]. Once license plate violation occurs, the system will be invalid.

**Figure 1:**
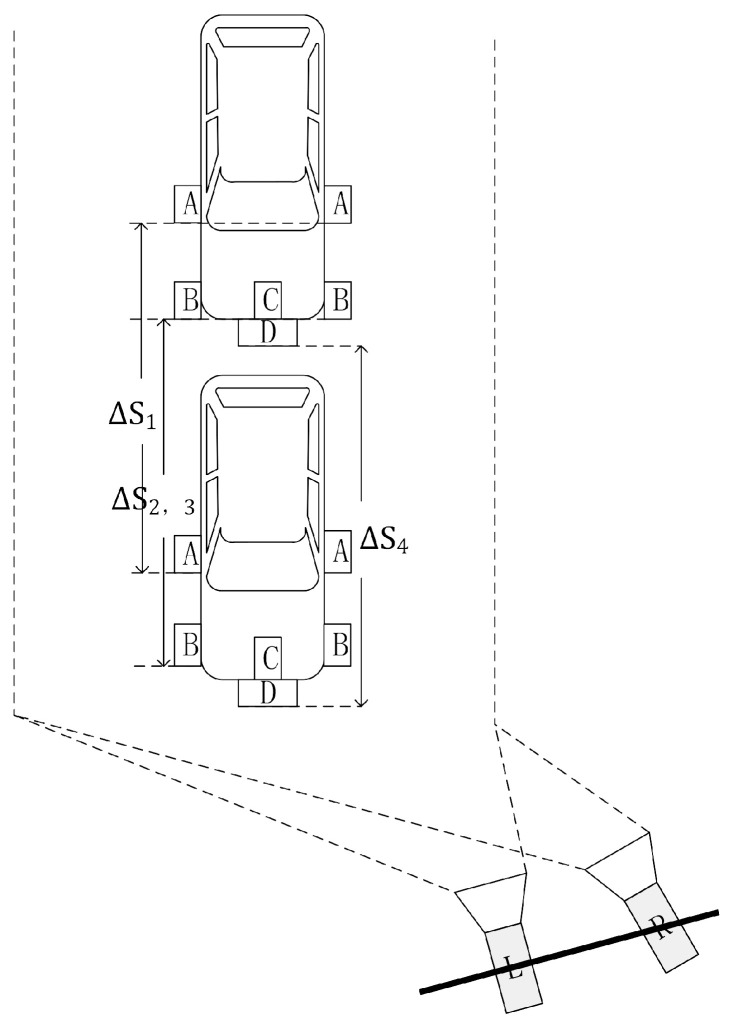
The proposed system model.

A robust binocular stereovision-based vehicle speed measurement system with vehicle multi-characteristic detection is proposed in this paper. The system model is shown in Figure 1, wherein, A, B, C and D respectively represent the multiple characteristics of vehicle, i.e., mirror, light, logo, and license plate. $\Delta S_{1}$, $\Delta S_{2}$, $\Delta S_{3}$, and $\Delta S_{4}$, respectively, represent the displacements of multiple characteristics in the speed measurement frame interval Δt. As the multiple characteristics of the vehicle are of multiple scales at multiple distances, You Only Look Once (YOLO) v4 network is selected, and a YOLOv4-based vehicle multi-characteristic detection network is proposed, which can efficiently detect multiple characteristics of vehicle. Combined with attention mechanism, Efficient Channel Attention (ECA) module is chosen, and an ECA-YOLOv4-based vehicle multi-characteristic detection network is proposed, which can further improve the detection efficiency and accuracy of the network for multiple characteristics of the vehicle. Accordingly, a multi-characteristic combined speed measurement system based on binocular stereovision with license plate, logo and light detection is proposed. In normal case, this system can efficiently enhance the speed measurement accuracy. In the case of license plate violation, this system can effectively improve the speed measurement robustness, which can solve the problem that the system in [14] cannot measure the vehicle speed in the case of license plate violation.

The rest of the paper is organized as follows. In Section 2, related work is discussed. In Section 3, a robust binocular stereovision-based vehicle speed measurement system with vehicle multi-characteristic detection is proposed. The main work focuses on the optimization of object detection algorithm for multiple characteristics of vehicle and the design of the multi-characteristic combined vehicle speed measurement system. In Section 4, the experimental setup and results are reported. In Section 5, the conclusion is drawn.

## 2. Related Works

### 2.1. Object Detection Algorithm

Accurate vehicle characteristic detection, namely, accurate object detection is an important premise of the vehicle speed measurement system proposed in this paper, and is also the main research content of this paper. Object detection includes two broad categories: traditional object detection and deep learning-based object detection [23]. Traditional object detection extracts informative feature sets by handcrafted features, while deep learning based object detection extracts informative feature sets by various end-to-end deep learning networks [24]. In recent years, thanks to the rapid development of artificial intelligence, object detection based on deep learning has become the mainstream, with better adaptability and more intelligence. Deep learning networks can be divided into Convolutional Neural Networks (CNNs) for Euclidean data and the currently state-of-the-art Graph Neural Networks (GNNs) for non-Euclidean data [25]. CNNs extract multi-scale local spatial information and fuse them to construct feature representation [26]. GNNs capture the dependency in graph by information transfer between nodes in graph, among which Graph Convolutional Networks (GCNs) have become a hot topic. An improved version of GCNs, known as miniGCNs, has been proposed, which allows to train in a minibatch fashion and is capable of inferring out-of-sample data without retraining [27]. However, as the object detection in this paper is performed on the captured stereo images which belong to Euclidean data, CNNs based object detection is enough for this application scene.

CNN-based object detection algorithm aims to detect the object of interest from the image, determine the category of each object, and locate the bounding box of each object by self-learning the high-level features of the images. Feature extraction backbone network includes AlexNet [28], VGG [29], Inception [30], ResNet [31], DenseNe [32], DarkNet [33], CSPDarkNet [34], and so on. According to the different ways of using the extracted feature maps, the object detection algorithms can be divided into three categories: object detection algorithm based on single feature map, object detection algorithm based on pyramid feature hierarchy, and object detection algorithm based on Feature Pyramid Network (FPN) [35].

R-CNN [36], Fast R-CNN [37], Faster R-CNN [38], and YOLOv1 [33] are all based on the single feature map. The advantage is the fast detection speed and little memory requirement. The disadvantage is that only the features of the last high-level are used; the resolution is low and is not conductive to small object detection.

SSD [15], RFB [39], and YOLOv2 [40] are all based on the pyramid feature hierarchy. The advantage is the simultaneous use of multi-layer feature maps. The disadvantage is that the calculated low-level high-resolution feature maps are not reused, the spatial information in the low-level feature maps is not fully utilized, and the small object detection task is still not well completed.

The FPN-based object detection algorithm takes the feature pyramid hierarchy as the foundation structure and makes joint prediction by the fused feature map of each layer. Commonly used feature fusion schemes include additive fusion, elementwise multiplicative fusion, and concatenation fusion [27]. Several state-of-the-art fusion strategies include concatenation-based fusion: early fusion, middle fusion, and late fusion, and compactness-based fusion: encoder–decoder fusion and cross-fusion [41]. The purpose of feature fusion is to combine the resulting features using different fusion strategies before the final classification. By feature fusion from the top layer to the bottom layer, the fused feature map contains both rich high-level semantic information and detailed low-level location information [42,43]. Then, the predicted joint feature pyramid hierarchy is used for object detection. For distinguishing multiple objects of multiple scales in the image, multiple feature maps in the predicted joint feature pyramid hierarchy can be used. YOLOv3 [44], M2Det [45], Retinanet [46], YOLOv4 [34], and EfficientDet [47] are all based on FPN. The advantage is that it can transfer the semantic information from the high-level to the low-level by information fusion and effectively supplement the semantic information to the low-level, therefore obtain features with high-resolution and rich semantic information, which can successfully complete the small object detection task.

### 2.2. Attention Mechanism

The essence of the attention mechanism is to locate the information of interest and suppress the useless information. In object detection network, the convolution operation module in the feature extraction backbone network can be replaced by the attention module to assign more weight to the part containing the information of interest and reduce the weight of the part containing the interference information. Thereby, the object detection accuracy can be improved.

The attention module can be divided into spatial attention module, channel attention module, and mixed attention module according to the attention principle [48,49,50]. The task of spatial attention module is to find the 2D spatial position containing the information of interest in a single feature map. It performs average pooling and maximum pooling on the single feature map of the input features, connects the average pooling, and maximum pooling results by convolution, and generates a corresponding 2D spatial attention features map.

The task of channel attention module is to find the channel position containing the information of interest among different channels. Squeeze-and-Excitation (SE) [51] and ECA [52] are typical representatives of channel attention module. SE performs global average pooling on the feature map of each channel, obtains the correlation between the two fully connected layers by sharing the multi-layer perception (MLP), and generates a corresponding channel attention map. For the two convolutions between the two fully connected layers in SE by which the channel dimension is first reduced and then increased, ECA replace them with a one-dimensional convolution that utilizes the local cross channel interaction information which fuses the information of each channel and its k neighbors. Dimension reduction can be avoided by cross-channel interaction which is a kind of information fusion as well. ECA can significantly reduce the model complexity while improving the network performance. It is an extremely lightweight channel attention module.

The task of mixed attention module is to simultaneously find the channel position containing the information of interest among different channels and the 2D spatial position containing the information of interest in each channel. Convolutional Block Attention Module (CBAM) [53] is a typical representative of mixed attention module. It divides the attention process into two independent cascaded modules: channel attention module and spatial attention module. First, channel attention map is generated by the channel attention module for input features, and the refined features of channel attention optimization are obtained by multiplying the input features with the generated channel attention map. Then, spatial attention map is generated by the spatial attention module for the refined feature, and the features of channel attention and spatial attention joint optimization are obtained by multiplying the refined features of channel attention optimization with the generated spatial attention map. Mixed attention model can refine features from both spatial and channel dimensions at the same time, which can better obtain the feature information of the region of interest and provide more effective information for prediction, thus improving the performance of object detection algorithm.

### 2.3. Cross-Entropy Loss

Cross-entropy is an important concept in Shannon’s information theory. Cross-entropy loss is an important index to measure the performance of an object detection classification model in deep learning training. It measures the similarity between the prediction and the actual target, which can effectively avoid the problem of learning rate decline in gradient descent. Cross-entropy loss increases as the predicted probability diverges from the actual label. For multi-class classification, the cross-entropy loss function is as shown in Equation (1)  [54].

(1) $$L=\frac{1}{N}\sum_{i=1}^{N}\left[-\sum_{j=1}^{C}y_{ij}\log\left(p_{ij}\right)\right]$$

wherein, *C* is the number of classes; *N* is the number of samples; $y_{ij}$ is the indicator variable, which is 1 if the predicted class of sample *i* is the same as the actual class *j*, and 0 otherwise; $p_{ij}$ is the predicted probability that the sample *i* belongs to the actual class *j*. The smaller the cross-entropy loss is, the better the model prediction will be.

## 3. The Proposed Method

**Figure 2:**
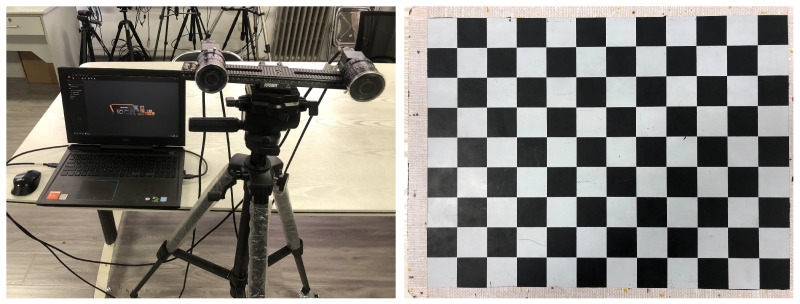
System configuration.

The system proposed is built by two industrial cameras and a laptop. The Hikvision MV-CA050-11UC industrial camera has a resolution of 2448 × 2048, with a Wallis WL1608-5MP fixed-focus lens of 8 mm. The laptop is equipped with an Intel Core i7-10750H CPU, 16 GB RAM, and a Nvidia RTX2060 6G graphics card. The stereo camera is calibrated by Zhengyou Zhang’s calibration method, and the cell size of the calibration board is 30 mm × 30 mm. The system configuration is shown in Figure 2.

**Figure 3:**
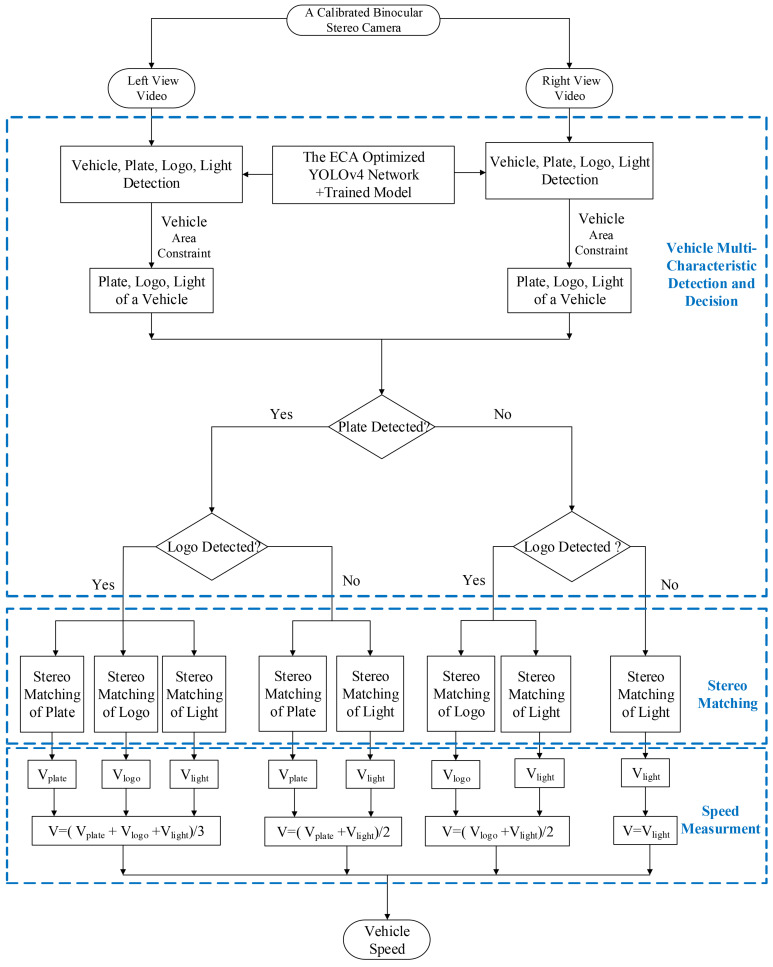
The whole procedure of the proposed vehicle speed measurement system with vehicle multi-characteristic detection.

The whole procedure of the proposed system is shown in Figure 3, which includes three parts: vehicle multi-characteristic detection and decision, stereo matching, and speed measurement. In the vehicle multi-characteristic detection and decision part, the proposed ECA-YOLOv4 object detection network is trained by the vehicle multi-characteristic dataset to get a model. The model is used to detect the left-view and right-view images separately to obtain multiple characteristics of vehicle, i.e., vehicle, license plate, logo, and light. The bounding box of the detected vehicle is used to constrain the detected multiple characteristics into the same vehicle area. Assuming the light characteristic always exists and can be detected, the speed measurement scheme is decided according to whether license plate and logo exist in the detection results, which can be divided into four cases: (1) three vehicle characteristics detected, i.e., license plate, logo and light detected; (2) two vehicle characteristics detected, i.e., license plate and light detected; (3) two vehicle characteristics detected, i.e., logo and light detected; (4) one vehicle characteristic detected, i.e., only light detected. In the stereo matching part, the stereo matching algorithm in [14] is reused, while the speed measurement point selection is slightly adjusted for the multiple characteristics with irregular shapes. In the speed measurement part, the binocular stereovision calibration algorithm in [14] is reused to perform speed measurement of single characteristic respectively, then the vehicle speed is calculated according to the speed measurement scheme decided in the detection and decision part.

### 3.1. Vehicle Multi-Characteristic Detection Based on YOLOv4

**Figure 4:**
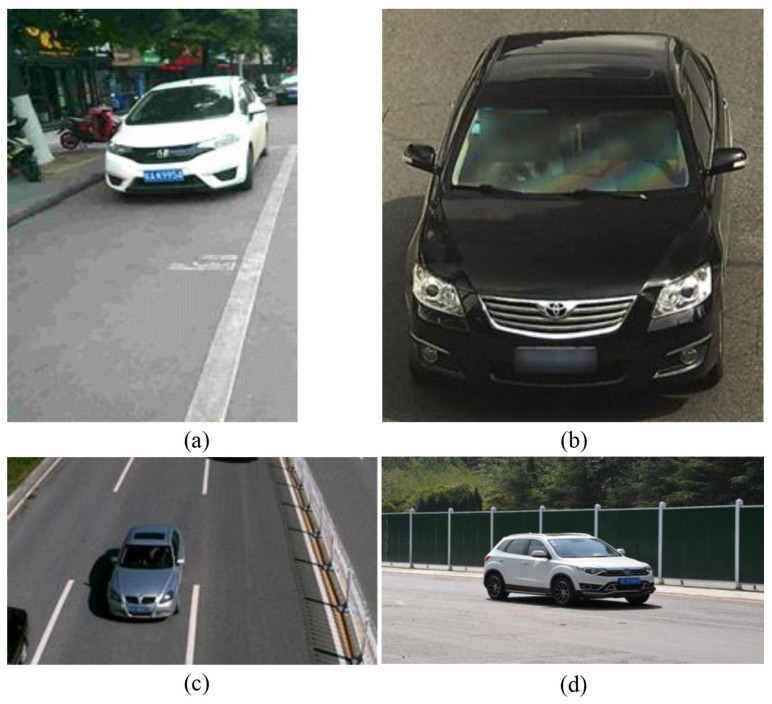
Image examples of our vehicle multi-characteristic dataset. (**a**) Image from CCPD. (**b**) Image from BIT Vehicle. (**c**) Image from Open ITS. (**d**) Self-captured image.

First, the vehicle multi-characteristic dataset is constructed. We randomly select 6103 images with a resolution of 1600 × 1200 from the Open ITS dataset [55], 1921 images with a resolution of 1920 × 1080 from the BIT Vehicle dataset [56], and 280 images with a resolution of 720 × 1160 from the CCPD dataset [57]. In addition, we capture 3351 images with a resolution of 6000 × 4000 by Nikon d3200 SLR camera and 480 images with a resolution of 2448 × 2048 by the Hikvision MV-CA050-11UC camera. The dataset has a total of 12,135 vehicle images with multiple characteristics. Figure 4 shows some image examples of our vehicle multi-characteristic dataset.

**Figure 5:**
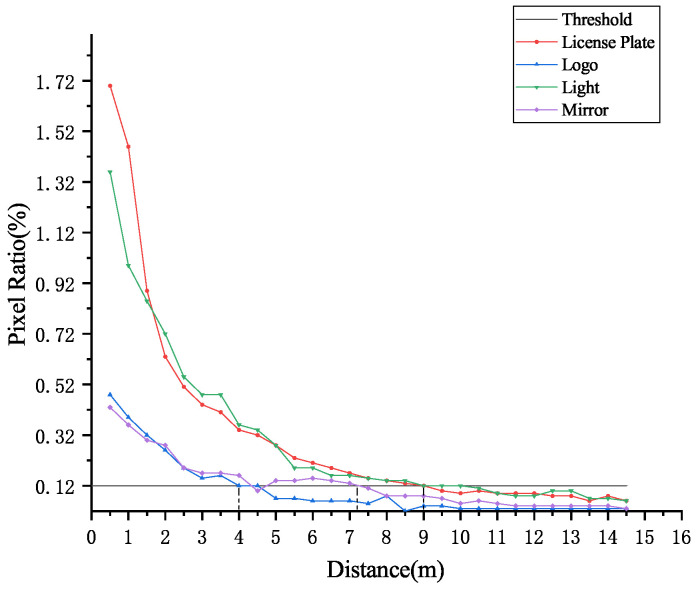
Pixel ratio graph of multiple characteristics in the speed measurement range of 0 to 15 m.

According to regulation on the relative size of small objects by SPIE, an object with a pixel ratio less than 0.12% can be regarded as small object, otherwise it can be regarded as regular object [58]. Statistical analysis is performed on the pixel ratio of four common vehicle characteristics (license plate, logo, light, and mirror) in the image captured at different distance within the speed measurement range. As shown in Figure 5, the distance threshold of small object for the license plate is about 9 m; the distance threshold of small object for the logo is about 4 m; the distance threshold of small object for the light is about 9 m; and the distance threshold of small object for the mirror is about 7 m. Therefore, the detection of multiple characteristics of vehicle is a multi-scale object detection problem.

**Figure 6:**
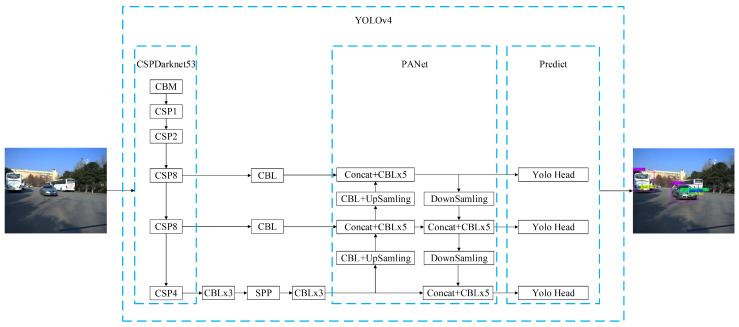
Schematic block diagram of the YOLOv4-based vehicle multi-characteristic object detection algorithm.

As mentioned above, object detection algorithms based on single feature map and pyramidal feature hierarchy are not suitable for this multi-scale varying object detection problem, while object detection algorithm based on FPN is suitable for this multi-scale varying object detection problem. Therefore, the object detection algorithm based on FPN is selected to detect the multiple characteristics of vehicle. Among them, YOLOv4 based on FPN is chosen due to its faster speed and higher accuracy [34]. Figure 6 is the schematic block diagram of the YOLOv4-based vehicle multi-characteristic object detection algorithm. Features are extracted from the input vehicle images by the feature extraction backbone network CSPDarknet53. The extracted features of different scales are fused by SPP and PANet. Finally, prediction is performed on the three different scale feature maps output by PANet, so as to obtain the bounding box, category, and confidence of the multiple characteristics of vehicle.

**Figure 7:**
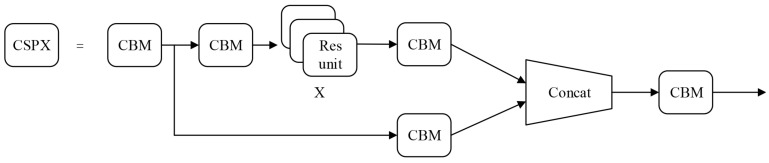
CSP structure in YOLOv4.

**Figure 8:**
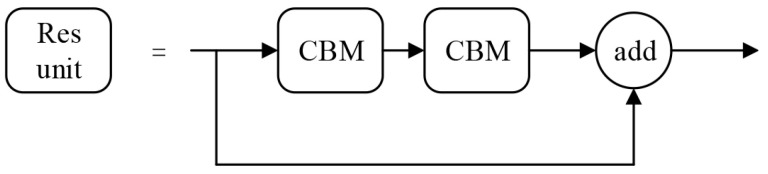
Residual unit structure in YOLOv4.

There are 23 Cross-Stage Partial (CSP) modules in CSPDarknet53. The CSP module enhances the learning ability of CNN and reduces the memory usage, whose structure is shown in Figure 7. The CBM module is composed of convolution, batch normalization and mish activation function. The structure of the residual unit is shown in Figure 8. The input feature map is added to the original feature map after two convolution operations.

After three convolution operations on the last feature map of CSPDarknet53, the Spatial Pyramid Pooling (SPP) module executes maximum pooling operations of four different scales, i.e., 1×1, 5×5, 9×9, and 13×13, and then the maximum pooling results of multiple scales are cascaded. After that, the feature maps are fused from the bottom to the top by PANet, and then the fused feature maps are fused again from the top to the bottom. Finally, the bounding box, category, and confidence of the object are predicted by the fused feature maps of three different scales.

The performance of the YOLOv4-based vehicle multi-characteristic detection algorithm is experimentally verified. Six representative object detection algorithms, i.e., Faster R-CNN, SSD, RFB, Retinanet, M2Det, and YOLOv3 are also selected for performance comparison. During the experiment, the model training parameters are the same, and the same model measurement indexes AP and mAP are selected. The experimental results are shown in Table 1. YOLOv4 has an AP of 96.47% for car, 92.13% for license plate, 87.72% for logo, 94.17% for light, and 91.2% for mirror, whose detection accuracy for vehicle single-characteristic detection is better than the other six algorithms. The average accuracy mAP of YOLOv4 is 92.34%, which is also better than that of the other six algorithms. Experimental results show that the proposed algorithm based on YOLOv4 is suitable for vehicle multi-characteristic detection and performs well.

### 3.2. Vehicle Multi-Characteristic Detection Based on ECA-YOLOv4

The vehicle characteristics to be detected are local information, and the attention mechanism that conforms to the human perception mechanism helps to focus on the local information. As mentioned above, the multiple vehicle characteristics to be detected are multi-scale varying, and a large proportion of them are small objects. Thus, the channel attention mechanism is suited to solve such problem. However, the position of the vehicle characteristics in the imaging plane constantly changes with the moving of the vehicle, and the spatial attention mechanism is not applicable. Therefore, the channel attention module is selected to optimize the YOLOv4-based vehicle multi-characteristic detection algorithm. In the two commonly used channel attention modules, the ECA module is an upgrade version of the SE module. Therefore, the ECA module is chosen to optimize the network structure of the YOLOv4 and improve the detection performance.

In the ECA module [52], cross-channel interaction and channel weight sharing are adopted. A weight matrix $W_{k}$ is defined for the cross-channel interaction, as shown in Equation (2).

(2) $$W_{k}=\left[\begin{array}{cccccccc} \omega_{1}^{1} & \cdots & \omega_{1}^{k} & 0 & 0 & \cdots & \cdots & 0\\ 0 & \omega_{2}^{2} & \cdots & \omega_{2}^{k+1} & 0 & \cdots & \cdots & 0\\ \vdots & \vdots & \vdots & \vdots & \ddots & \vdots & \vdots & \vdots \\ 0 & \cdots & 0 & 0 & \cdots & \omega_{C}^{C-k+1} & \cdots & \omega_{C}^{C} \end{array}\right]$$

wherein $W_{k}$ has k×C parameters. For the ith channel $y_{i}$, as shown in Equation (3), the interactive relationship of *k* adjacent channels $y_{i}^{j}$ (including $y_{i}$) to $y_{i}$ should be considered to calculate its weight $\omega_{i}$, j=1,…,k.

(3) $$\omega_{i}=\sigma \left(\sum_{j=1}^{k}\omega_{i}^{j}y_{i}^{j}\right),\;y_{i}^{j}\in \Omega_{i}^{k}$$

wherein $\Omega_{i}^{k}$ represents the set is composed of the *k* adjacent channels of $y_{i}$

After cross-channel interaction, channel weight sharing is carried out, so that all the channels share the same k parameters, as shown in Equation (4).

(4) $$\omega_{i}^{\prime }=\sigma \left(\sum_{j=1}^{k}\omega^{j}y_{i}^{j}\right),\;y_{i}^{j}\in \Omega_{i}^{k}$$

In this way, the parameter number of the weight matrix $W_{k}$ are reduced from k×C to *k*. Weight parameter learning can be achieved by 1D fast convolution with a convolution kernel size of *k*.

(5) $$\omega =\sigma \left(C1D_{k}\left(y\right)\right)$$

wherein C1D represents 1D convolution operation.

**Figure 9:**
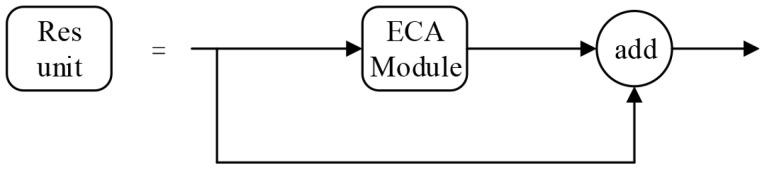
Residual unit structure in ECA-YOLOv4.

As shown in Figure 9, the two CBM modules in each residual unit in each CSP module of YOLOv4 are replaced by one ECA module, and the YOLOv4 is improved to ECA-YOLOv4. Moreover, SE channel attention module and CBAM mixed attention module are also, respectively, used to improve YOLOv4 to SE-YOLOv4 and CBAM-YOLOv4 in the same way for performance comparison. The model parameter sizes and floating point operations (FLOPs) of the object detection networks with different attention modules are shown in Table 2. For the same input size of 416×416, the model parameter size of YOLOv4 is 244.29 M, the model parameter size of CBAM-YOLOv4 is 180.51 M with a compression rate of 26.10%, the model parameter size of SE-YOLOv4 is 179.66 M with a compression rate of 26.45%, and the model parameter size of ECA-YOLOv4 is 178.82 M with a compression rate of 26.8%. ECA-YOLOv4 has the smallest model parameter size. For the same input size of 416×416, the FLOPs of YOLOv4 is 29.95 G, the FLOPs of CBAM-YOLOv4 is 19.28 G, the FLOPs of SE-YOLOv4 is 19.28 G, and the FLOPs of ECA-YOLOv4 is 19.28 G. ECA-YOLOv4, SE-YOLOv4, and CBAM-YOLOv4 have the same FLOPs, which is smaller than that of YOLOv4.

During the experiment, the model training parameters are the same, and the same model measurement indexes AP, mAP, and FPS are selected. The experimental results are shown in Table 3. From the AP of vehicle, license plate, logo, and mirror, the detection performances of CBAM-YOLOv4, SE-YOLOv4, and ECA-YOLOv4 are all superior to that of YOLOv4. From the AP of light, the detection performances of SE-YOLOv4 and ECA-YOLOv4 are superior to that of YOLOv4. From the mAP, the detection performances of CBAM-YOLOv4, SE-YOLOv4, and ECA-YOLOv4 are all superior to that of YOLOv4. From the FPS, only the detection speed of ECA-YOLOv4 is faster than that of YOLOv4. Note that for all four networks, the AP of logo is slightly lower than that of other vehicle characteristics. This is because the shapes of logos are more diverse than those of license plates, lights and mirrors, and a few special logos may not be accurately detected, which will reduce the precision corresponding to some recall and thus reduce the AP of logo.

Considering the four indexes of model parameter size, AP, mAP, and FPS. ECA-YOLOv4 has the smallest model parameter size and the fastest FPS, with AP for vehicle single-characteristic detection and mAP for vehicle multi-characteristic superior to that of YOLOv4 and equivalent to that of CBAM-YOLOv4, and SE-YOLOv4. In summary, the performance of ECA-YOLOv4 is better than that of the other three algorithms.

### 3.3. Optimal Design of Binocular Stereovision-Based Vehicle Speed Measurement System with Vehicle Multi-Characteristic Detection

**Figure 10:**
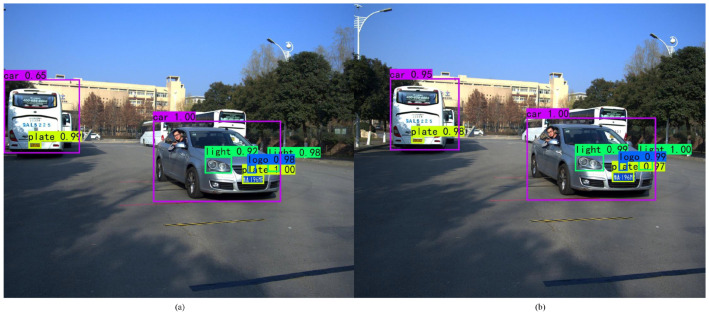
Vehicle multi-characteristic detection results: (**a**) left-view and (**b**) right-view.

After the multiple characteristics of the vehicle are detected, as shown in Figure 10, stereo matching is performed on each detected vehicle characteristic by the stereo matching algorithm in [14]. Thus, the stereo matching point pairs in the left-view and right-view images of the vehicle characteristic are obtained. Then, the matching point pair with the smallest square sum of the Euclidean distance to the bounding box center is selected as the speed measurement point. Zhengyou Zhang’s calibration method is used to calculate the 3D coordinates of the selected speed measurement point, the displacement of adjacent speed measurement points are calculated, and the vehicle speed can be obtained by dividing the displacement by the time interval.

**Figure 11:**
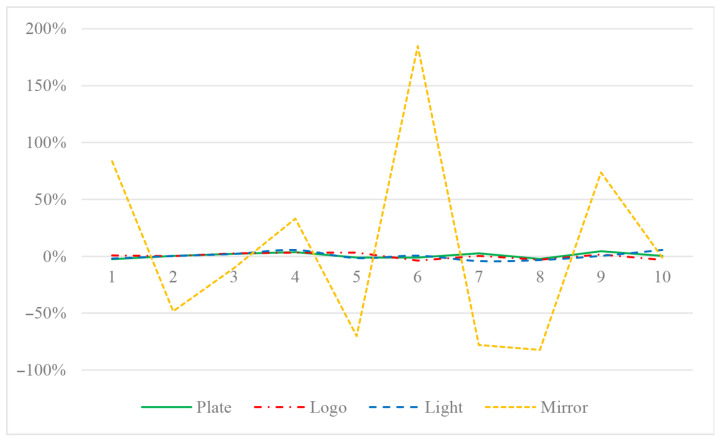
Speed measurement error rate curve for single characteristic of vehicle.

The video capture frame rate is 30FPS, the vehicle speed is set to 43 km/h, the vehicle drives to the camera in a straight line from far and near at a constant speed, and the speed measurement is performed ten times per second. The speed measurement data by a professional satellite speed meter P-Gear P-510 are taken as the ground truth for comparison. The speed measurement results of single characteristic are shown in Table 4, and the error rate curve is shown in Figure 11. It can be seen from Table 4 that the speeds measured by the license plate, logo, and light are all consistent with the satellite ground truth. The error rate range is [−2.49%, 4.55%], [−3.71%, 3.20%], and [−4.16%, 5.66%], respectively, all of which do not exceed the 6% error rate limit specified by the China national standard GB/T21255-2007 [59]. However, the speeds measured by the mirror differs greatly from the satellite ground truth, with an error rate range of [−82.27%, 184.69%], which is far more than the 6% error rate limit.

By analyzing the speed measurement procedure of the mirror, the large error is mainly due to the smooth surface which is lack of detailed information such as texture. Thus, large error occurs in the stereo matching, which leads to the incorrect speed measurement. Therefore, in the following design of the multi-characteristic speed measurement system, only three vehicle characteristics (license plate, logo, and light) are selected, and the mirror characteristic is abandoned.

Assuming that the vehicle light characteristic always exists, the vehicle speed measurement algorithm of the proposed binocular stereovision vehicle speed measurement system with vehicle multi-characteristic detection is designed as Algorithm 1.
**Algorithm 1** Vehicle Speed Measurement Algorithm**Input: ** Binocular Stereovision Video Sequence
**Output: ** Vehicle Speed
  1:  **function**
*Vehicle speed measurement*
  2:        **if** License plate detected **then**
  3:            **if** Logo detected **then**
  4:                 *v* = $\frac{v_{plate}+v_{logo}+v_{light}}{3}$
  5:            **else** Logo undetected
  6:                 *v* = $\frac{v_{plate}+v_{light}}{2}$
  7:            **end if**
  8:        **else** License plate undetected
  9:             **if** Logo detected **then**
10:                 *v*=$\frac{v_{logo}+v_{light}}{2}$
11:             **else** Logo undetected
12:                 *v* = $v_{light}$
13:             **end if**
14:        **end if**
15:        **return** *v*
16:  **end function**


## 4. Experiments

**Figure 12:**
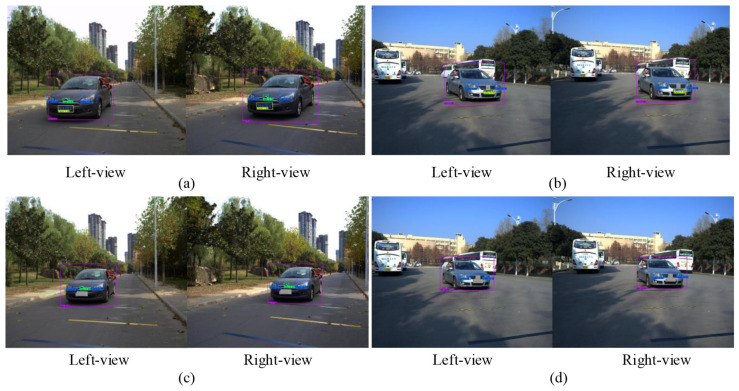
Four speed measurement scenarios. (**a**) Speed measurement scenario where license plate, logo and light all exist; (**b**) Speed measurement scenario where license plate and light exist; (**c**) Speed measurement scenario where logo and light exist; (**d**) Speed measurement scenario where only light exists.

In the actual speed measurement test, the video capture frame rate is 30 FPS, the vehicle drives to the camera in a straight line from far and near at a constant speed, and the speed measurement is performed ten times per second. The speed measurement data by the professional satellite speed meter P-Gear P-510 is taken as the ground truth for comparison. To verify the algorithm, four speed measurement scenarios are set up, as shown in Figure 12. Figure 12a,b shows the vehicle speed measurement experiments with license plate detected, while Figure 12c,d shows the vehicle speed measurement experiments with license plate undetected.

### 4.1. Vehicle Speed Measurement Experiments with License Plate Detected

First, experiments are carried out in the speed measurement scenario where license plate, logo, and light all exist, as shown in Figure 12a. Table 5 shows the speed measurement results of a single experiment in this scenario, in which the vehicle drives in a straight line at a constant speed of 46 km/h.

As shown in Table 5, the speed measurement error rate range is [−3.49%, 1.50%] for the license plate, [−4.07%, 5.01%] for the logo, [−5.97%, 2.28%] for the light, and [−3.53%, 1.52%] for the average of all the three characteristics of vehicle. The average speed measurement of all the three vehicle characteristics can effectively reduce the error rate fluctuation range of the individual speed measurement by individual vehicle characteristic. Multiple experiments are also carried out in this scenario, and the speed measurement results are shown in Table 6. The vehicle speeds set for test 2–8 are 44 km/h, 36 km/h, 32 km/h, 38 km/h, 40 km/h, 43 km/h, and 30 km/h, respectively. The error rate ranges of test 2–8 are [−3.08%, 2.92%], [−1.36%, 2.62%], [−2.23%, 3.61%], [−1.39%, 2.87%], [−2.04%, 3.97%], [−2.86%, 4.12%], and [−0.84%, 3.20%], respectively. The maximum absolute error rate is 4.12%, which meets the 6% error rate limit specified by the China national standard GB/T21255-2007 [59].

Next, as shown in Figure 12b, the logo is artificially covered to simulate the speed measurement scenario where license plate and light exist but logo does not exist, and the experiments are carried out therein. Table 7 shows the speed measurement results of a single experiment in this scenario, in which the vehicle drives in a straight line at a constant speed of 30 km/h.

As shown in Table 7, the speed measurement error rate range is [−4.76%, 5.22%] for the license plate, [−4.35%, 5.90%] for the light, and [−1.97%, 4.12%] for the average of the two characteristics of vehicle. The average speed measurement of the two vehicle characteristics can reduce the error rate fluctuation range of the individual speed measurement by individual vehicle characteristic. Multiple experiments are also carried out in this scenario, and the speed measurement results are shown in Table 8. The vehicle speeds set for test 2–8 are 45 km/h, 36 km/h, 43 km/h, 38 km/h, 46 km/h, 33 km/h, and 31 km/h, respectively. The error rate ranges of test 2–8 are [−4.16%, 4.50%], [−3.36%, 3.33%], [−2.76%, 4.58%], [−2.08%, 4.24%], [−4.02%, 1.84%], [−2.14%, 3.37%], and [−1.57%, 4.22%], respectively. The maximum absolute error rate is 4.58%, which also meets the 6% error rate limit specified by the China national standard GB/T21255-2007 [59].

### 4.2. Vehicle Speed Measurement Experiments with License Plate Undetected

Then, as shown in Figure 12c, the license plate is artificially covered to simulate the speed measurement scenario where logo and light exist but license plate does not exist, and the experiments are carried out therein. Table 9 shows the speed measurement results of a single experiment in this scenario, in which the vehicle drives in a straight line at a constant speed of 45 km/h.

As shown in Table 9, the speed measurement error rate range is [−3.20%, 4.38%] for the logo, [−4.72%, 4.84%] for the light, and [−2.79%, 2.30%] for the average of the two characteristics of vehicle. The average speed measurement of the two vehicle characteristics can reduce the error rate fluctuation range of the individual speed measurement by individual vehicle characteristic. Multiple experiments are also carried out in this scenario, and the speed measurement results are shown in Table 10. The vehicle speeds set for test 2–8 are 43 km/h, 36 km/h, 32 km/h, 38 km/h, 46 km/h, 33 km/h, and 31 km/h, respectively. The error rate ranges of test 2–8 are [−5.61%, 4.33%], [−2.99%, 3.93%], [−1.39%, 3.45%], [−3.85%, 3.84%], [−3.63%, 1.77%], [−2.50%, 4.99%], and [−1.64%, 4.62%], respectively. The maximum absolute error rate is 5.61%, which also meets the 6% error rate limit specified by the China national standard GB/T21255-2007 [59].

Finally, as shown in Figure 12d, the license plate and the logo are artificially covered to simulate the speed measurement scenario where only light exists but license plate and logo do not exist, and the experiments are carried out therein. Table 11 shows the speed measurement results of a single experiment in this scenario, in which the vehicle drives in a straight line at a constant speed of 36 km/h.

As shown in Table 11, the speed measurement error rate range is [−4.68%, 5.30%] for the light. Multiple experiments are also carried out in this scenario, and the speed measurement results are shown in Table 12. The vehicle speeds set for test 2–8 are 31 km/h, 32 km/h, 38 km/h, 46 km/h, 33 km/h, 30 km/h, and 45 km/h, respectively. The error rate ranges of test 2–8 are [−4.35%, 5.90%], [−3.87%, 4.57%], [−4.86%, 4.16%], [−5.97%, 2.28%], [−2.13%, 5.21%], [−3.90%, 4.67%], and [−4.72%, 4.84%], respectively. The maximum absolute error rate is 5.97%, which still meets the 6% error rate limit specified by the China national standard GB/T21255-2007 [59].

### 4.3. Contrast Experiments

The speed measurement results of the proposed system are compared with that of the original system in [14] in the same speed measurement scenario, including the speed measurement scenario with license plate not covered and the speed measurement scenario with license plate covered. The former is to verify the speed measurement accuracy of the proposed system, while the latter is to verify the speed measurement robustness of the proposed system.

**Figure 13:**
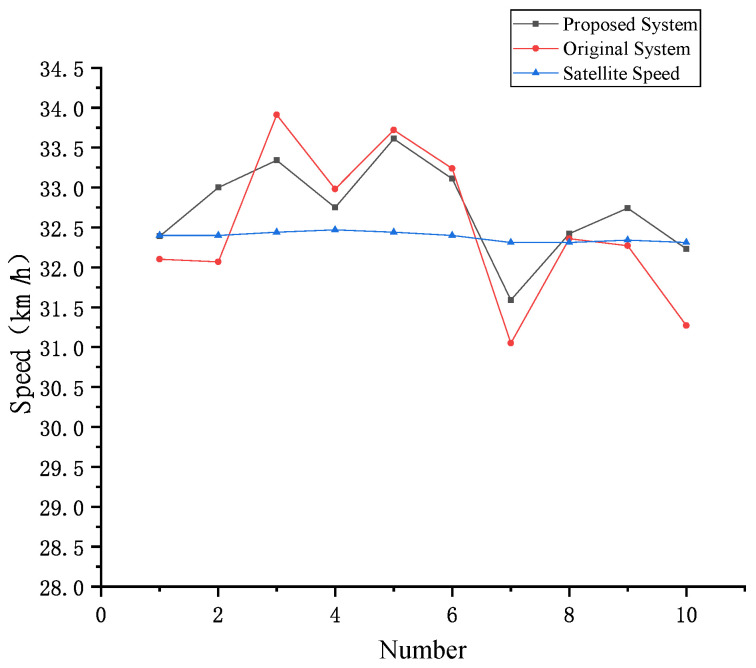
Speed measurement curve of two contrast experiments in the scenario with license plate not covered.

Table 13 shows the speed measurement results of two contrast experiments in the scenario with license plate not covered, in which the vehicle drives in a straight line at a constant speed of 32 km/h. Figure 13 is the corresponding speed measurement curve. As shown in Table 13, in the scenario with license plate not covered, the speed measurement error rate range of the proposed system is [−2.23%, 3.61%] and the maximum absolute error rate is 3.61%, while the speed measurement error rate range of the system in [14] is [−3.91%, 4.53%] and the maximum absolute error rate is 4.53%. Both meet the 6% error rate limit specified by the China national standard GB/T21255-2007 [59]. However, both the error rate range and the maximum absolute error rate of the proposed system are smaller than those of the system in [14]. As can be seen from the speed measurement curve in Figure 13, the proposed system has a smaller error fluctuation range, which is closer to the satellite ground truth, and has higher speed measurement accuracy.

Meanwhile, the speed measurement performances are compared between the proposed system and the various existing speed measurement methods. Table 14 shows a comparison of the speed measurement error between the proposed system and the other four methods in the scenario with license plate not covered. It can be seen that the root mean square error (RMSE) of the proposed system is smaller than that of the other four methods, and the maximum error is also smaller than that of the other four methods. Therefore, in the scenario with license plate not covered, the speed measurement accuracy of the proposed method is superior to that of the other four methods, that is, the speed measurement accuracy of the system is improved.

Table 15 shows the speed measurement results of two contrast experiments in the scenario with license plate covered, in which the vehicle drives in a straight line at a constant speed of 33 km/h. As shown in Table 15, in the scenario with license plate covered, the speed measurement error rate range of the proposed system is [−2.50%, 4.99%] and the maximum absolute error rate is 4.99%, which meets the 6% error rate limit specified by the China national standard GB/T21255-2007 [59], while the system in [14] fails in the speed measurement. The proposed system can still measure the vehicle speed accurately in the scenario with license plate covered.

Meanwhile, the speed measurement performances are compared between the proposed system and the various existing speed measurement methods. Table 16 shows a comparison of the speed measurement error between the proposed system and the other four methods in the scenario with license plate covered. It can be seen that the methods in [8,14] fail, and the RMSE and maximum error of the proposed system are smaller than that of the other two methods. Therefore, in the scenario with license plate covered, the speed measurement robustness of the proposed method is superior to that of the other two invalid methods, and the speed measurement accuracy of the proposed method is superior to that of the other two valid methods, that is, the speed measurement robustness of the system is improved.

## 5. Conclusions

In this study, we solved the problem of effectively measuring the vehicle speed based on binocular stereovision system in case of license plate violation. We proposed a robust binocular stereovision-based vehicle speed measurement system with vehicle multi-characteristic detection. We optimized the object detection algorithm for vehicle multi-characteristic detection, and thus optimized the whole vehicle speed measurement system. The FPN-based YOLOv4 object detection algorithm was selected for the vehicle multi-characteristic detection, and the ECA channel attention mechanism was combined to improve YOLOv4. An improved ECA-YOLOv4 object detection algorithm was proposed for the vehicle multi-characteristic detection, which was trained and verified by our constructed vehicle multi-characteristic dataset. The experimental results showed that the proposed ECA-YOLOv4 object detection algorithm can efficiently improve the detection accuracy of multiple characteristics of the vehicle and minimize the network model parameter size. Three vehicle characteristics, that is, license plate, logo, and light, were chosen to design the corresponding speed measurement system for vehicle multi-characteristic detection. Many experiments were carried out in different speed measurement scenarios. Experimental results show that the proposed speed measurement system improves the speed measurement accuracy efficiently and effectively solve the problems in robustness of the system in [14].

## Figures and Tables

**Table 1:** Performance comparison of multiple object detection algorithms for vehicle multi-characteristic detection.

Algorithm	AP${}_{\mathrm{car}}$ (%)	AP${}_{\mathrm{plate}}$ (%)	AP${}_{\mathrm{logo}}$ (%)	AP${}_{\mathrm{light}}$ (%)	AP${}_{\mathrm{mirror}}$ (%)	mAP (%)
Faster R-CNN	87.75	45.82	53.91	79.32	68.84	67.13
SSD	94.90	77.16	71.68	87.79	80.82	82.47
RFB	94.62	56.93	60.21	82.67	80.08	74.90
Retinanet	93.97	33.14	51.61	73.59	56.80	61.82
M2Det	95.87	68.75	71.15	87.27	84.84	81.58
YOLOv3	93.91	88.06	81.94	90.89	86.99	88.35
YOLOv4	96.47	92.13	87.72	94.17	91.20	92.34

**Table 2:** The model parameter size and FLOPs of the object detection networks with different attention modules.

Algorithm	Input Size	Model Parameters Size	Model Compression Rate	FLOPs
YOLOv4	416 × 416	244.29 MB	/	29.95 G
CBAM-YOLOv4	416 × 416	180.51 MB	26.10%	19.28 G
SE-YOLOv4	416 × 416	179.66 MB	26.45%	19.28 G
ECA-YOLOv4	416 × 416	178.82 MB	26.80%	19.28 G

**Table 3:** Performance comparison of the object detection networks with different attention modules.

Algorithm	AP${}_{\mathrm{car}}$ (%)	AP${}_{\mathrm{plate}}$ (%)	AP${}_{\mathrm{logo}}$ (%)	AP${}_{\mathrm{light}}$ (%)	AP${}_{\mathrm{mirror}}$ (%)	mAP (%)	FPS
YOLOv4	96.47	92.13	87.72	94.17	91.20	92.34	22
ECA-YOLOv4	97.01	93.19	87.98	94.53	91.99	92.94	23
SE-YOLOv4	97.02	93.24	88.30	94.49	92.29	93.07	21
CBAM-YOLOv4	97.27	93.47	88.48	93.91	91.76	92.98	16

**Table 4:** Speed measurement results of single characteristics of the vehicle.

Number	Plate		Logo		Light		Mirror		Satellite Speed (km/h)
	Speed (km/h)	Error Rate (%)	Speed (km/h)	Error Rate (%)	Speed (km/h)	Error Rate (%)	Speed (km/h)	Error Rate (%)	
1	42.29	−2.49	43.70	0.76	42.53	−1.94	79.65	83.65	43.37
2	43.55	0.41	43.41	0.08	43.50	0.30	22.43	−48.28	43.37
3	44.52	2.28	44.71	2.71	44.47	2.16	39.10	−10.18	43.53
4	45.32	3.56	45.16	3.20	46.21	5.60	58.24	33.09	43.76
5	43.28	−1.10	45.16	3.20	43.09	−1.53	13.04	−70.20	43.76
6	43.22	−1.06	42.06	−3.71	43.94	0.60	124.35	184.68	43.68
7	44.63	2.53	43.64	0.26	41.72	−4.16	9.57	−78.02	43.53
8	42.39	−2.25	42.05	−3.04	41.95	−3.27	7.69	−82.27	43.37
9	45.34	4.55	44.04	1.56	43.56	0.44	75.34	73.71	43.37
10	43.09	0.34	41.62	−3.07	45.37	5.66	42.48	−1.07	42.94
Error rate range	[−2.49%, 4.55%]		[−3.71%, 3.20%]		[−4.16%, 5.66%]		[−82.27%, 184.69%]		

**Table 5:** The speed measurement results in the scenario where license plate, logo, and light all exist.

Number	Plate		Logo		Light		Average		Satellite Speed (km/h)
	Speed (km/h)	Error Rate (%)	Speed (km/h)	Error Rate (%)	Speed (km/h)	Error Rate (%)	Speed (km/h)	Error Rate (%)	
1	44.81	−2.48	45.30	−1.41	44.64	−2.85	44.92	−2.25	45.95
2	45.79	−0.74	46.50	0.80	46.57	0.95	46.29	0.34	46.13
3	47.08	1.50	48.27	4.08	45.34	−2.24	46.90	1.11	46.38
4	47.02	1.01	48.88	5.01	45.87	−1.46	47.26	1.52	46.55
5	47.42	1.50	46.71	−0.02	43.93	−5.97	46.02	−1.50	46.72
6	47.21	1.41	44.67	−4.04	47.61	2.28	46.50	−0.12	46.55
7	46.87	0.48	45.64	−2.14	46.17	−1.01	46.23	−0.89	46.64
8	45.22	−3.39	46.36	−0.96	46.54	−0.58	46.04	−1.64	46.81
9	45.26	−3.49	46.25	−1.39	44.77	−4.54	45.43	−3.14	46.9
10	45.16	−3.33	44.82	−4.07	45.23	−3.19	45.07	−3.53	46.72
Error rate range	[−3.49%, 1.50%]		[−4.07%, 5.01%]		[−5.97%, 2.28%]		[−3.53%, 1.52%]		

**Table 6:** The error rates of multiple experiments in the average speed measurement of the three vehicle characteristics (license plate, logo, and light).

Number	Error Rate of Test 2 (%)	Error Rate of Test 3 (%)	Error Rate of Test 4 (%)	Error Rate of Test 5 (%)	Error Rate of Test 6 (%)	Error Rate of Test 7 (%)	Error Rate of Test 8 (%)
1	−3.08	0.25	−0.01	1.55	−2.04	−1.22	−0.77
2	−1.14	1.93	1.84	2.82	−1.39	0.27	1.90
3	2.92	1.20	2.77	1.80	0.86	2.38	−0.84
4	0.47	−1.28	0.87	1.96	−1.07	4.12	−0.28
5	−1.91	0.84	3.61	−0.88	1.24	0.19	3.20
6	1.02	1.89	2.20	2.06	0.26	−1.39	1.65
7	1.61	−0.88	−2.23	2.87	−1.55	−0.46	1.06
8	−2.77	1.88	0.33	−1.39	−0.51	−2.86	0.78
9	−2.23	−1.36	1.25	0.36	−0.04	2.18	1.07
10	1.65	2.62	−0.24	1.54	3.97	0.98	1.65
Error rate range	[−3.08%, 2.92%]	[−1.36%, 2.62%]	[−2.23%, 3.61%]	[−1.39%, 2.87%]	[−2.04%, 3.97%]	[−2.86%, 4.12%]	[−0.84%, 3.20%]

**Table 7:** The speed measurement results in the scenario where license plate and light exist.

Number	Plate		Light		Average		Satellite Speed (km/h)
	Speed (km/h)	Error Rate (%)	Speed (km/h)	Error Rate (%)	Speed (km/h)	Error Rate (%)	
1	28.84	−3.62%	30.92	3.35%	29.88	−0.14%	29.92
2	29.97	2.62%	30.57	4.68%	30.27	3.65%	29.2
3	30.58	1.83%	31.36	4.42%	30.97	3.12%	30.03
4	31.80	5.22%	30.96	2.44%	31.38	3.83%	30.22
5	31.04	1.44%	29.62	−3.20%	30.33	−0.88%	30.6
6	31.12	1.69%	29.72	−2.87%	30.42	−0.59%	30.6
7	30.91	0.41%	29.45	−4.35%	30.18	−1.97%	30.79
8	29.05	−4.76%	32.30	5.90%	30.67	0.57%	30.5
9	29.78	−2.68%	30.35	−0.81%	30.07	−1.74%	30.6
10	32.01	4.51%	31.77	3.74%	31.89	4.12%	30.63
Error rate range	[−4.76%, 5.22%]		[−4.35%, 5.90%]		[−1.97%, 4.12%]		

**Table 8:** The error rates of multiple experiments in the average speed measurement of the two vehicle characteristics (license plate and light).

Number	Error Rate of Test 2 (%)	Error Rate of Test 3 (%)	Error Rate of Test 4 (%)	Error Rate of Test 5 (%)	Error Rate of Test 6 (%)	Error Rate of Test 7 (%)	Error Rate of Test 8 (%)
1	−3.02	1.53	−2.21	1.96	−2.67	−0.57	0.14
2	−1.55	2.11	0.36	2.67	0.11	−1.95	1.48
3	4.50	1.32	2.22	0.76	−0.37	1.41	−1.57
4	−1.48	−3.36	4.58	3.53	−0.23	−0.57	−0.02
5	−2.24	0.92	−1.31	−2.08	−2.24	1.95	2.58
6	0.42	1.63	−0.23	4.24	1.84	1.03	0.19
7	2.89	0.33	−0.82	2.54	−0.26	−0.87	4.22
8	−2.99	1.54	−2.76	−0.68	−1.99	1.53	1.07
9	−4.16	−0.26	2.49	−1.44	−4.02	−2.14	3.25
10	1.02	3.33	3.00	2.67	−3.26	3.37	1.95
Error rate range	[−4.16%, 4.50%]	[−3.36%, 3.33%]	[−2.76%, 4.58%]	[−2.08%, 4.24%]	[−4.02%, 1.84%]	[−2.14%, 3.37%]	[−1.57%, 4.22%]

**Table 9:** The speed measurement results in the scenario where logo and light exist.

Number	Logo		Light		Average		Satellite Speed (km/h)
	Result (km/h)	Error Rate (%)	Result (km/h)	Error Rate (%)	Result (km/h)	Error Rate (%)	
1	42.96	−3.20	43.59	−1.78	43.28	−2.49	44.38
2	44.61	−0.34	44.47	−0.65	44.54	−0.49	44.76
3	44.73	−0.24	47.01	4.84	45.87	2.30	44.84
4	46.98	4.38	44.75	−0.59	45.87	1.90	45.01
5	44.52	−1.26	43.14	−4.32	43.83	−2.79	45.09
6	46.09	2.22	44.40	−1.54	45.25	0.34	45.09
7	44.68	−0.94	46.44	2.97	45.56	1.02	45.1
8	44.04	−2.32	44.28	−1.80	44.16	−2.06	45.09
9	45.83	1.65	42.96	−4.72	44.40	−1.54	45.09
10	46.44	2.90	45.28	0.33	45.86	1.62	45.13
Error rate range	[−3.20%, 4.38%]		[−4.72%, 4.84%]		[−2.79%, 2.30%]		

**Table 10:** The error rates of multiple experiments in the average speed measurement of the two vehicle characteristics (logo and light).

Number	Error Rate of Test 2 (%)	Error Rate of Test 3 (%)	Error Rate of Test 4 (%)	Error Rate of Test 5 (%)	Error Rate of Test 6 (%)	Error Rate of Test 7 (%)	Error Rate of Test 8 (%)
1	−1.38	0.51	0.45	1.85	−2.13	−2.50	−0.02
2	−0.03	0.97	3.26	2.36	0.88	0.67	3.57
3	2.27	1.40	1.90	2.48	0.92	2.38	−1.64
4	1.29	−0.91	0.52	0.01	1.77	−1.11	−0.11
5	0.21	2.29	3.45	−1.16	−3.00	2.52	4.31
6	0.01	1.45	1.99	0.93	−0.88	−1.26	4.62
7	4.33	−2.99	−1.39	3.84	−1.58	−0.61	−0.82
8	−5.61	3.93	0.42	−3.85	−0.77	0.22	1.24
9	−0.81	−1.08	1.98	0.76	−2.96	1.02	−0.02
10	0.36	2.03	1.25	−0.11	−3.63	4.99	0.62
Error rate range	[−5.61%, 4.33%]	[−2.99%, 3.93%]	[−1.39%, 3.45%]	[−3.85%, 3.84%]	[−3.63%, 1.77%]	[−2.50%, 4.99%]	[−1.64%, 4.62%]

**Table 11:** The speed measurement results in the scenario where only light exists.

Number	Light (km/h)	Satellite Speed (km/h)	Error Rate (%)
1	37.31	36.12	3.30
2	36.24	36.11	0.37
3	36.86	36.19	1.84
4	34.56	36.26	−4.68
5	37.75	36.33	3.90
6	36.67	36.49	0.49
7	35.22	36.19	−2.68
8	38.17	36.25	5.30
9	36.72	36.21	1.41
10	37.26	36.22	2.87
Error rate range	[−4.68%, 5.30%]		

**Table 12:** The error rates of multiple experiments in the speed measurement of the one vehicle characteristic (light).

Number	Error Rate of Test 2 (%)	Error Rate of Test 3 (%)	Error Rate of Test 4 (%)	Error Rate of Test 5 (%)	Error Rate of Test 6 (%)	Error Rate of Test 7 (%)	Error Rate of Test 8 (%)
1	3.35	1.82	2.96	−2.85	−0.03	2.56	−1.78
2	4.68	4.57	1.61	0.95	1.61	4.39	−0.65
3	4.42	−1.23	1.07	−2.24	5.01	−3.90	4.84
4	2.44	0.74	1.21	−1.46	−0.15	0.59	−0.59
5	−3.20	4.41	−3.82	−5.97	5.21	4.18	−4.32
6	−2.87	2.10	4.16	2.28	−1.25	4.67	−1.54
7	−4.35	−0.03	4.16	−1.01	1.67	3.64	2.97
8	5.90	−3.87	−4.86	−0.58	5.03	2.28	−1.80
9	−0.81	4.14	−2.46	−4.54	−2.13	3.25	−4.72
10	3.74	4.33	0.51	−3.19	4.81	0.19	0.33
Error rate range	[−4.35%, 5.90%]	[−3.87%, 4.57%]	[−4.86%, 4.16%]	[−5.97%, 2.28%]	[−2.13%, 5.21%]	[−3.90%, 4.67%]	[−4.72%, 4.84%]

**Table 13:** Speed measurement results of two contrast experiments in the scenario with license plate not covered.

Number	Proposed System (km/h)			System in [14] (km/h)			Satellite Speed (km/h)
	Speed (km/h)	Error (km/h)	Error Rate (%)	Speed (km/h)	Error (km/h)	Error Rate (%)	
1	32.39	0.01	−0.03	32.10	−0.30	−0.94	32.4
2	33.00	0.60	1.84	32.07	−0.33	−1.02	32.4
3	33.34	0.90	2.77	33.91	1.47	4.53	32.44
4	32.75	0.28	0.87	32.98	0.51	1.56	32.47
5	33.61	1.17	3.61	33.72	1.28	3.94	32.44
6	33.11	0.71	2.20	33.24	0.84	2.61	32.4
7	31.59	−0.72	−2.23	31.05	−1.26	−3.91	32.31
8	32.42	0.11	0.33	32.36	0.05	0.15	32.31
9	32.74	0.40	1.25	32.27	−0.07	−0.21	32.34
10	32.23	−0.08	−0.24	31.27	−1.04	−3.21	32.31
Error rate range	[−2.23%, 3.61%]			[−3.91%, 4.53%]			

**Table 14:** Error comparison among different speed measurement methods in the scenario with license plate not covered.

	RMSE (km/h)	Max Error (km/h)
Luvizon et al. [8]	1.36	[−4.68, +6.00]
Tang et al. [60]	6.59	NA
VSS-SURF [61]	1.29	[−2.0, +2.0]
System in [14]	0.65	[−1.6, +1.1]
Proposed System	0.43	[−0.72, +1.17]

**Table 15:** Speed measurement results of two contrast experiments in the scenario with license plate covered.

Number	Proposed System (km/h)			System in [14] (km/h)			Satellite Speed (km/h)
	Speed (km/h)	Error (km/h)	Error Rate (%)	Speed (km/h)	Error (km/h)	Error Rate (%)	
1	32.96	−0.84	−2.50	Invalid	Invalid	Invalid	33.8
2	33.79	0.22	0.67	Invalid	Invalid	Invalid	33.56
3	34.57	0.81	2.38	Invalid	Invalid	Invalid	33.76
4	33.39	−0.37	−1.11	Invalid	Invalid	Invalid	33.76
5	34.61	0.85	2.52	Invalid	Invalid	Invalid	33.76
6	33.24	−0.42	−1.26	Invalid	Invalid	Invalid	33.66
7	33.36	−0.20	−0.61	Invalid	Invalid	Invalid	33.56
8	33.48	0.08	0.22	Invalid	Invalid	Invalid	33.4
9	33.70	0.34	1.02	Invalid	Invalid	Invalid	33.36
10	34.91	1.66	4.99	Invalid	Invalid	Invalid	33.25
Error rate range	[−2.50%, 4.99%]			NA			

**Table 16:** Error comparison among different speed measurement methods in the scenario with license plate covered.

	RMSE (km/h)	Max Error (km/h)
Luvizon et al. [8]	Invalid	Invalid
Tang et al. [60]	6.59	NA
VSS-SURF [61]	1.29	[−2.0, +2.0]
System in [14]	Invalid	Invalid
Proposed System	0.48	[−0.84, +1.66]

## Data Availability

Not applicable.
